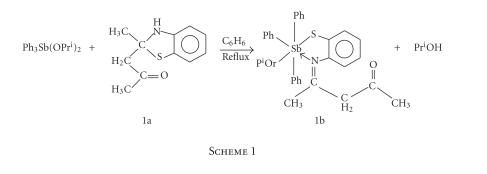# Antispermatogenic Activity of the Benzothiazoline Ligand and Corresponding Organoantimony(V) Derivative in Male Albino Rats

**DOI:** 10.1155/BCA/2006/16895

**Published:** 2006-04-18

**Authors:** Pankaj K. Sharma, H. Rehwani, A. K. Rai, R. S. Gupta, Y. P. Singh

**Affiliations:** ^1^Department of Chemistry, University of Rajasthan, Jaipur 302 004, India; ^2^Reproduction Physiology Section, Department of Zoology, University of Rajasthan, Jaipur 302 004, India

## Abstract

Triphenylantimony(V) derivative, Ph_3_Sb(OPr^i^) [SC_6_H_4_N : C(CH_3_)CH_2_C(O)CH_3_], **1b**, and the corresponding benzothiazoline ligand [1, 2], $\text{H}\overset{⎴}{{\text{NC}}_{6}{\text{H}}_{4}\text{SC}}({\text{CH}}_{3}){\text{CH}}_{2}\text{C}(\text{O}){\text{CH}}_{3}$, **1a**, have been tested for their effects on the reproductive system of male albino rats. The oral administration of both **1a** and **1b** at the dose level of 10 mg/rat/day produced significant reduction in the weights of testes, epididymides, seminal vesicles, and ventral prostate. Significant decrease in sperm motility as well as in sperm density resulted in 100% sterility. Significant (*P* < .01) alterations were also found in biochemical parameters of reproductive organs in treated male rats as compared to the control group. Production of preleptotene, pachytene, and secondary spermatocytes was decreased by 42%, 43%, 39%, and by 44%, 49%, 55% in the ligand, **1a**, and organoantimony(V) derivative, **1b**, treated rats, respectively. These results indicate that both compounds **1a** and **1b** are antispermatogenic in nature and on oral administration in male rats, and finally caused sterility. A comparison indicates that the organoantimony(V) derivative **1b** is more effective pertaining to its antispermatogenic activity than the corresponding ligand **1a**.

## INTRODUCTION

Organic compounds containing −NC_6_H_4_S− unit are well known for their significant biological activities [3]. Phenothiazenes significantly affect the hypothalamous pituitary gonadal axis, resulting in a delay in ovulation and menstruation in women [3]. These effects have also been observed in rats and dogs [4, 5]. The rate of implantation was lowered, and reduction in litter size has been reported by some phenothiazine derivatives [6, 7]. In general biological activity of such type of compounds enhances considerably on complexation with metal atom [8]. We have earlier reported the antifertility activity of organoantimony(III) [9] and aluminium(III) [10] derivatives of benzothiazolines. In respect of the reproductive and developmental toxicity, antimony compounds have also been studied in experimental model: no teratogenic effects were found, when pregnant ewes and rats were treated with trivalent antimony potassium tartrate (2 mg/Kgbt) and antimony trichloride (0.1 and 1 mg/dl), respectively [11, 12]. Antimony had quite widespread use in pharmacology for the treatment of syphilis, fever, melancholy, pneumonia, epilepsy, and inflammatory conditions [13]. Organic antimony salts are used medically to treat some tropical diseases [14], especially in the treatment of all forms of leishmenasis [15]. Organoantimony compounds also exhibit significant antimicrobial [16] as well as antitumor activities [17, 18], which is associated with cytostatic activity [19] similar to that for cisplatin. The biological toxicity of these derivatives is much less than that for Pt and Pd anticancer substances [19, 20]. A large number of antimony(III) compounds have also been tested as bactericides [21] and fungicides [22].

A survey of literature revealed that so far no attention has been paid to compare the effects of benzothiazoline ligand with its metal derivatives on the reproductive system of male rats. In view of this, we have synthesized and characterized the benzothiazoline ligand and its organoantimony(V) derivative. In the present publication, a comparative study among the effects on the reproductive systems of male albino rats of the corresponding ligand, **1a**, and its organoantimony(V) derivative, **1b**, versus control animals is presented, and also a comparison of the effects of these compounds **1a** and **1b** is made pertaining to their antispermatogenic activities.

## EXPERIMENTAL

In view of the moisture sensitive nature of the starting materials, all the synthetic reactions were performed under moisture free conditions. All the chemicals used were of reagent grade. Solvents (E Merck) were dried by standard methods before use. Ph_3_SbBr_2_ [23] and triphenylantimony(V) isopropoxide [24] were prepared by literature methods. The benzothiazoline ligand $\text{H}\overset{⎴}{{\text{NC}}_{6}{\text{H}}_{4}\text{SC}}({\text{CH}}_{3}){\text{CH}}_{2}\text{C}(\text{O}){\text{CH}}_{3}$, **1a**, was prepared by the reported method [1, 2] and was purified by distillation under vacuum (85–88°C, 0.1 atm) before use. Organoantimony(V) derivative, **1b**, of the benzothiazoline was prepared (Scheme 1) by the reported method [25]. Antimony was estimated iodometrically [26]. Nitrogen and sulphur were estimated by Kjeldahl's and Messenger's methods, respectively [26]. Isopropanol and isopropoxide were estimated by the chromate oxidimetric method [27]. Molecular weight of the organoantimony(V) derivative, **1b**, was determined ebullioscopically using Beckmann's thermometer. IR spectra of these compounds were recorded on a Nicolet DX FTIR spectrophotometer in the range 4000–200 cm^−1^ on a CsI cell. ^1^H and ^13^C NMR spectra were recorded in DMSO-d_6_ solution on a JEOL-FX-90Q (90 MHz) or Brucker DPX-300 MHz spectrometer, using TMS as an internal and external references, respectively.

## BIOLOGICAL TESTS

Sexually mature male albino rats of laboratory bred, Wistar strain weighing about 150–175 g (90–100 days old) were used in the experiments. They were acclimatized to the normal laboratory conditions of light-dark cycle (12L:12D) with the temperature around 20 ± 5°C and 35%–60% relative humidity. Animals were given standard rat diet [Ashirwad Industries Ltd] and water ad libitum throughout the study.

The animals were randomly allocated into three experimental groups of six rats each. In the first control group, only olive oil (0.5 mL/rat/day) was orally administered for 60 days. In the second and third groups, oral administration of the corresponding ligand, **1a**, and its organoantimony(V) derivative, **1b**, was given in olive oil at the same doses (10 mg/rat/day) for 60 days.

The fertility test of each experimental male rat was assessed by natural mating with two pro-estrous and virgin females, before, during, and after days 55 to 60 of the treatment. The presence of sperm cells in the vaginal smears was accepted as evidence of copulation. Mated females were separated and then allowed to complete the term. The number of litters delivered was recorded and used as an index for fertility of the males. Body weights of the experimental rats were monitered throughout the study. All experimental males were sacrificed under light ether anaesthesia, approximately 24 hours following the last dose. Final body weights of the animals were recorded. Blood samples were collected by cardiac puncture and serum was separated by centrifugation. Testosterone was determined by Radio Immuno Assay. Epididymal sperm motility and number of spermatozoa in the epididymides and testes were determined by the method of Prasad et al [28]. The testes, epididymides, and other accessory sex organs were excised and freed from the surrounding fat and connecting tissues and weighed. Biochemical estimations of protein, sialic acid, glycogen, cholesterol, and fructose [29–33] were carried out in testes, epididymides, and other accessory sex organs. For histopathological examination, tissues were fixed in Bouin's fluid and several sections of the testes were prepared and stained by means of haematoxylin and eosin. Testicular cell population dynamics was performed by using “Camera Lucida” drawing. Mean seminiferous tubular diameter was determined. Various testicular cell components were quantitatively analyzed [34]. Difference between groups were compared by using one way analysis of variance (ANOVA), followed by the individual paired “T test.” Differences were considered to be statistically significant when *P* < .01. All data are presented as mean ± SEM.

## RESULTS AND DISCUSSION

Triphenylantimony(V) derivative, **1b**, of the benzothiazoline ligand, **1a**, has been synthesized (Scheme 1) and characterized by the reported method [25].

The light brown colored, viscous compound, **1b**, is soluble in common organic solvents. Ebullioscopic molecular weight measurement reveals its monomeric nature in benzene solution as reported earlier [25].

## ANTIFERTILITY ACTIVITY

Oral administration of corresponding ligand, **1a**, and its antimony derivative, **1b**, reduce fertility in treated rats. The body weight of the rats treated with these compounds (**1a** and **1b**) was not affected during the course of the experiments. However, the weights of testes, epididymides, seminal vesicles, and ventral prostate were reduced significantly (*P* < .01) in **1a** and **1b** treated rats than those in the control group (Table 1). Motility of spermatozoa, removed from the cauda epididymides of the treated rats (**1a** and **1b**), was highly depressed when compared with control animals (Table 2). Sperm density in testes and in cauda epididymides was depleted significantly (*P* < .01) in both treated groups (group II and III) as compared to controls (Table 2).

Significant loss of sperm motility and density give rise to 100% sterility in **1a** and **1b** treated rats. Testosterone level in both the treated groups reduced significantly (Table 2). Suppressive effects of these compounds were noticed (Table 3) in protein content and sialic acid content of testes, epididymides, and other accessory sex organs. Testicular glycogen content and fructose content of seminal vesicles were also decreased, whereas testicular cholesterol was elevated in this investigation.

The ligand, **1a**, and its corresponding metal derivative, **1b**, used in this investigation resulted in weight loss of testes and other accessory sex organs mainly due to hormone deficiency. Testes produce the male gametes and a site of spermatogenesis. Spermatogenesis is regulated by pituitary hormones (FSH, LH), secreted into the peripheral circulation and by androgen, synthesized and secreted in close proximity to target sites within the testes [35]. Thus testes, epididymides, and other accessory sex organs are androgen-dependent for their growth and function. Reduction in weights may reflect a declined amount and synthesis of androgen within these organs [36]. Decrease in sperm motility and density could compromise the fertility [37]. Low sperm concentration is associated with low fertility. The spermatozoa can utilize glucose as well as fructose [38, 39]. Fructose is the main source of energy required by spermatozoa. The results from this study indicate that these compounds (**1a** and **1b**) decrease the fructose level, since the inhibition of fructose and the decrease in sperm motility were always correlated [40]. Immotility of sperm may be due to structural defects of the flagellum, for example, axonemal microtubular abnormalities or defective mitochondria [41–43].

The results demonstrate a marked decreases in testicular glycogen. Such glycolytic inhibition may explain the reduced sperm motility observed in vitro in the absence of lactate and pyruvate [44, 45]. A marked decrease in the glycogen content could affect protein synthesis and thus subsequently inhibit spermatogenesis [46]. The integrity and functional activity of sperm membrane are crucial for viability and, also, for the physiological changes that occur at the sperm surface during the fertilization process including capacitation, acrosome reaction, and binding to the zona pellucida and oolemma [47]. Sialic acids are concerned with changing the membrane surface of maturing spermatozoa and with the development of their fertilizing capacity [48]. Thus decreased sialic acid may inhibit the fertilizing capacity of sperm.

 The production of preleptotene spermatocytes, pachytene spermatocytes, and secondary spermatocytes was decreased by 42%, 43%, and 39%, respectively, in **1a** treated rats and by 44%, 49%, and 55%, respectively, in **1b** treated rats. The total number of Sertoli cells and seminiferous tubular diameter were also reduced in **1a** and **1b** treated rats as compared to the control group (Table 4). Sertoli cells decreased significantly in these series of experiments. Sertoli cells synthesized and secreted ABP's (androgen binding proteins) that are believed to serve as a reservoir for testosterone and maintaining the high intratubular concentrations, necessary for completion of spermatogenesis [49]. Alteration in the Sertoli cells affect the production of ABP which in turn lead to inhibition of the spermatogenesis [50]. The effect of metal administration produces unmistakable damage to the Sertoli cells [51]. Reduction in the number of spermatogonia, spermatocytes, and spermatids may indicate lower availability of FSH and LH, which are essential for initiation and maintainance of spermatogenesis. Cholesterol is a precursor for androgen biosynthesis and its level in testes is closely related to fertility and sperm output. Accumulation of cholesterol indicates its reduced conversion into the androgen [52]. It is known that sperm production cannot proceed optimally to completion without a continuous androgen supply [53].

From the above results it may be concluded that the benzothiazoline ligand, **1a**, and its corresponding organoantimony(V) derivative, **1b**, used in this investigation, are able to reduce fertility in male rats possibly by interfering the process of spermatogenesis, and it is found that the compound **1b** has more suppressive effects on male reproductive systems as compared to its corresponding ligand, **1a**. These results are in close agreement with the earlier reports on the enhanced activity of metal complexes in comparison to the parent ligand [8].

## Figures and Tables

**Table 1:** Effects of compounds **1a** and **1b** on body and organs weight in male rats.

Treatment	Final body weight (g)	Organs weight (mg/100 gbwt)			
		Testes	Epididymides	Seminal vesicles	Ventral prostate

Group-I	230±5.65	1390±20.50	640.25 ± 24	690.40 ± 16.80	475.100 ± 12.5
Control					
Group-II	197.5^ns^ ± 27.5	1286.52* ± 24.76	524.57* ± 30.83	582.84* ± 32.20	299.14** ± 4.44
P1 treated					
Group-III	187.5^ns^ ± 12.5	1208.18* ± 56.39	466.80** ± 10.91	554.17** ± 4.67	265.85** ^a^ ± 6.85
P2 treated					

All values are expressed as mean ± SE, ns: nonsignificant

Level of significance * *P* < .01; * *P* < .001 compared to control group

^a^ *P* < .01; ^b^ *P* < .001 compared to P1 treated group.

**Table 2:** Effect of compounds **1a** and **1b** on sperm motility and number in male rats.

Treatment	Sperm motility (%)	Sperm density (million/mL)		Fertility (%)	Testosterone ng/dL
	Cauda epididymides	Testes	Cauda epididymides		

Group-I	68.00 ± 1.10	4.10 ± 0.45	45.45 ± 0.95	100 %(+ve)	5.25 + 0.05
Control					
Group-II	27.16** ± 0.84	2.65* ± 0.22	12.65** ± 1.15	100 %(−ve)	2.40 + 0.48*
**1a** treated					
Group-III	21.12** ^a^ ± 0.96	1.90* ± 0.36	8.40** ^a^ ± 0.86	100 % (−ve)	1.02 + 0.12**
**1b** treated					

All values are expressed as mean ± SE

Level of significance * *P* < .01; ** *P* < .001 compared to control group

^a^ *P* < .01 compared to **1a** treated group.

**Table 3:** Effect of compounds **1a** and **1b** on biochemical parameters in male rats.

Treatment	Protein (mg/g)				Sialic acid (mg/g)				Glycogen (mg/g)	Cholesterol (mg/g)	Fructose (mg/g)

	Testes	Cauda epididymides	Seminal vesicle	Ventral prostate	Testes	Cauda epididymides	Ventral prostate	Seminal vesicle	Testes	Testes	Seminal vesicle

Group-I	244.05	224.40	212.45	208.0	5.18	6.05	5.45	5.68	3.40	5.28	4.65
Control	±3.65	±2.98	±3.50	±2.05	±0.12	±0.08	±0.10	±0.18	±0.18	±0.51	±0.10
Group-II	185.35**	200.6**	198.60**	182.0**	4.42*	5.74**	4.75**	5.18**	2.48*	12.53*	3.00**
**1a** treated	±2.05	±0.80	±1.05	±2.02	±0.2	±0.11	±0.09	±0.02	±0.22	±1.62	±0.11
Group-III	167.00**	190.28** ^a^	191.25** ^a^	179.5**	4.14**	5.27**	4.47*	4.80* ^b^	2.16*	14.18*	2.77**
**1b** treated	±4.42	±3.05	1.65	±1.88	±0.10	±0.08	±0.20	±0.05	±0.19	±2.08	±0.25

All values are expressed as mean ± SE

Level of significance * *P* < .01; ** *P* < .001 compared to control group.

^a^ *P* < .01; ^b^ *P* < .001 compared to **1a** treated group.

**Table 4:** Effect of compounds **1a** and **1b** on testicular cell population dynamics.

Treatment	Testicular cell counts (number/10 cross-section)					Seminiferous tubular diameter (*μ*m)

	Sertoli cell	Spermatogonia	Preleptotene spermatocyte	Pachytene spermatocyte	Secondary sprermatocyte	

Group-I	2.79 ± 0.05	6.97 ± 0.77	22.82 ± 1.11	36.42 ± 1.37	48.50 ± 2.85	250.80 ± 6.68
Control						
Group-II
P1 treated	2.45* ± 0.12	5.89 ± 0.03	13.25* ± 1.30	20.56** ± 0.70	29.55* ± 1.27	215.25* ± 1.72
Percent	(−12%)	(−15%)	(−42%)	(−43%)	(−39%)	(−14%)
deviation ^E^
Group-III
P2 treated	2.32** ± 0.02	5.92 ± 0.72	14.80** ± 0.17	18.61** ± 1.33	21.93** ^a^ ± 2.59	176.09* ± 3.03
Percent	(−17%)	(−15%)	(−44%)	(−49%)	(−55%)	(−30%)
deviation^E^

All values are expressed as mean ± SE

Level of significance * *P* < .01; ** *P* < .001 compared to control group.

^a^ *P* < .01; ^b^ *P* < .001 compared to **1a** treated group.

^E^Values in parentheses are percentage reduction in particular cell type.

**Scheme 1:**